# Competition between pentoses and glucose during uptake and catabolism in recombinant *Saccharomyces cerevisiae*

**DOI:** 10.1186/1754-6834-5-14

**Published:** 2012-03-16

**Authors:** Thorsten Subtil, Eckhard Boles

**Affiliations:** 1 Institute of Molecular Biosciences, Goethe-University Frankfurt am Main, Max-von-Laue-Str. 9, D-60438 Frankfurt am Main, Germany

**Keywords:** Hexokinase, Glucose, Arabinose, Xylose, Fermentation, Lignocellulose, Pentose, Ethanol, *Saccharomyces*, Yeast

## Abstract

**Background:**

In mixed sugar fermentations with recombinant *Saccharomyces cerevisiae *strains able to ferment D-xylose and L-arabinose the pentose sugars are normally only utilized after depletion of D-glucose. This has been attributed to competitive inhibition of pentose uptake by D-glucose as pentose sugars are taken up into yeast cells by individual members of the yeast hexose transporter family. We wanted to investigate whether D-glucose inhibits pentose utilization only by blocking its uptake or also by interfering with its further metabolism.

**Results:**

To distinguish between inhibitory effects of D-glucose on pentose uptake and pentose catabolism, maltose was used as an alternative carbon source in maltose-pentose co-consumption experiments. Maltose is taken up by a specific maltose transport system and hydrolyzed only intracellularly into two D-glucose molecules. Pentose consumption decreased by about 20 - 30% during the simultaneous utilization of maltose indicating that hexose catabolism can impede pentose utilization. To test whether intracellular D-glucose might impair pentose utilization, hexo-/glucokinase deletion mutants were constructed. Those mutants are known to accumulate intracellular D-glucose when incubated with maltose. However, pentose utilization was not effected in the presence of maltose. Addition of increasing concentrations of D-glucose to the hexo-/glucokinase mutants finally completely blocked D-xylose as well as L-arabinose consumption, indicating a pronounced inhibitory effect of D-glucose on pentose uptake. Nevertheless, constitutive overexpression of pentose-transporting hexose transporters like Hxt7 and Gal2 could improve pentose consumption in the presence of D-glucose.

**Conclusion:**

Our results confirm that D-glucose impairs the simultaneous utilization of pentoses mainly due to inhibition of pentose uptake. Whereas intracellular D-glucose does not seem to have an inhibitory effect on pentose utilization, further catabolism of D-glucose can also impede pentose utilization. Nevertheless, the results suggest that co-fermentation of pentoses in the presence of D-glucose can significantly be improved by the overexpression of pentose transporters, especially if they are not inhibited by D-glucose.

## Background

The cost effective production of fuels and chemicals from plant biomass requires efficient conversion of all sugars present in the raw materials. While cornstarch or sugarcane hydrolyzates mainly consist of hexoses, hydrolyzates from lignocellulosic biomass also contain pentose sugars like D-xylose or L-arabinose. The yeast *Saccharomyces cerevisiae*, traditionally used for industrial ethanol production lacks the ability to ferment pentoses. However, intensive research and genetic engineering approaches during the last years improved the capability of *S. cerevisiae *for pentose utilization.

D-xylose fermentation by *S. cerevisiae *was first achieved by expression of a xylose reductase (XR) and a xylitol dehydrogenase (XDH) from *Scheffersomyces stipitis *[1]. However differences in co-factor specificities of the two enzymes can result in co-factor imbalances, production of varying amounts of xylitol and therefore reduced ethanol yields [1-4]. Another strategy used heterologous expression of genes encoding xylose isomerases. Fungal as well as bacterial xylose isomerases could be functionally expressed and enabled the yeast cells to ferment D-xylose efficiently [5-8].

L-arabinose utilization in *S. cerevisiae *has been achieved by introducing multi-step oxidoreductive fungal or multi-step bacterial pathways. Similarly to the fungal D-xylose utilization pathway, the fungal L-arabinose pathway employs NADPH- and NADH-dependent redox reactions resulting in severe co-factor imbalances [9] which could be avoided by introduction of bacterial L-arabinose pathways [10,11].

The expression of pentose converting enzymes in *S. cerevisiae *is not sufficient for optimal pentose fermentation. Overexpression of xylulokinase or genes of the non-oxidative part of the pentose phosphate pathway turned out to be beneficial as well as overexpression of uptake systems able to transport pentoses [12-15]. Nevertheless, co-consumption of pentoses with D-glucose is still limited and normally *S. cerevisiae *does not utilize pentoses before D-glucose depletion. This has been attributed to competiton of the sugars during their uptake into the cells. Pentoses like D-xylose and L-arabinose are taken up by *S. cerevisiae *cells via their hexose uptake systems [14,16]. Due to the preference of the transporters for D-glucose, pentose uptake is competitively inhibited by D-glucose. Unfortunately, up to now no specific pentose transporters could be functionally expressed in yeast which specifically mediate uptake of only pentoses into *S. cerevisiae *cells but are not able to transport hexoses or are not inhibited by D-glucose [15,17-21]

It has never been analyzed whether D-glucose inhibits pentose utilization only during sugar uptake or whether also D-glucose metabolism impairs the simultaneous utilization of pentose sugars. Here we describe the impact of intracellular D-glucose as well as extracellular D-glucose and D-glucose metabolism on pentose utilization in yeast strains expressing bacterial D-xylose and L-arabinose pathways. We used maltose as a non-competitive carbon source as well as hexo-/glucokinase deletion strains to selectively block D-glucose catabolism. Our analyses demonstrate that sugar uptake is the main competitive step to impair co-consumption of pentoses together with D-glucose. Moreover, we demonstrate that overexpression of pentose-transporting hexosetransporters like Hxt7 and Gal2 partially relieves the inhibitory effect of D-glucose on pentose utilization.

## Results

### The influence of hexose catabolism on pentose utilization in recombinant *S. cerevisiae *cells

It has been shown that pentoses like D-xylose and L-arabinose are taken up by *S. cerevisiae *cells via their hexose uptake systems [12,14,21,22]. However, the yeast hexose transporters generally have lower affinities for pentoses than for hexoses [16]. Therefore, it is believed that co-consumption of D-glucose and pentoses is impaired due to the preference of the uptake systems for D-glucose and its competition with pentose sugars. Nevertheless, it has never been investigated whether the further steps of pentose catabolism might also be impaired by the simultaneous catabolism of D-glucose. D-glucose and pentose catabolism share all the enzymes from glycolysis starting with phosphofructokinase.

To distinguish between inhibitory effects of D-glucose on pentose uptake and further pentose catabolism we used maltose as an alternative carbon source in maltose-pentose co-consumption experiments. Maltose is taken up by specific maltose permeases [23,24] and only hydrolyzed intracellularly by maltases into two D-glucose molecules which are then normally channeled into the glycolytic pathway by hexo-/glucokinases. Therefore, unlike D-glucose maltose utilization does not compete with pentose utilization at the transport level. On the other hand, as transcription of most of the yeast hexose transporters which are able to transport pentoses with sufficient capacities [14] must normally be induced by glucose or galactose, such transporters had to be overexpressed constitutively in order to ensure sufficient pentose uptake.

Recombinant D-xylose and L-arabinose utilizing strains BWY1-XT and BWY1-AT, respectively, were constructed. Strain BWY1 is derived from CEN.PK2-1 C and has been obtained by evolutionary engineering for improved L-arabinose utilization after expression of genes for a bacterial L-arabinose metabolizing pathway [10,11]. For D-xylose utilization BWY1 was transformed with plasmids overexpressing a codon-optimized version of the *Clostridium phytofermentans *xylose isomerase gene [6], the xylulokinase gene of the fungus *Hypocrea jecorina *and the yeast hexose transporter gene *HXT7 *which has been shown to be able to support uptake of D-xylose [14], resulting in BWY1-XT. For L-arabinose utilization BWY1 was transformed with plasmids overexpressing an optimized bacterial L-arabinose catabolic pathway [11] and the yeast D-galactose transporter gene *GAL2 *which has been shown to be able to support uptake of L-arabinose [21], resulting in BWY1-AT. The strains BWY1-XT and BWY1-AT were able to use D-xylose or L-arabinose, respectively, as the sole carbon and energy sources.

**Figure 1:**
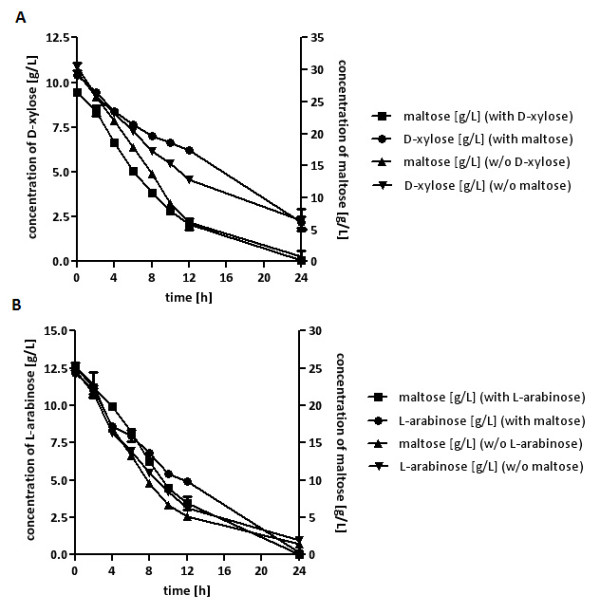
**Co-consumption of maltose and pentose sugars**. **A) **The strain BWY1-XT was inoculated with 4 g _(dry biomass)_/L in SC medium containing D-xylose (10 g/L) without or with 30 g/L maltose. **B) **The strain BWY1-AT was inoculated with 4 g _(dry biomass)_/L in SC medium containing L-arabinose (12 g/L) without or with 25 g/L maltose. The cultivations were performed in shake flasks at 30°C. At different time points, probes were taken and the concentrations of residual sugars analyzed by HPLC.

Cells of strains BWY1-XT or BWY1-AT were pregrown in D-xylose or L-arabinose synthetic complete (SC) media and inoculated in SC media with D-xylose or L-arabinose in the presence or absence of maltose (Figure 1). High cell density fermentations (about 4 g _(dry biomass)_/L) were performed to increase sugar consumption. The pentoses were consumed simultaneously with the maltose. Nevertheless, the maximal D-xylose consumption rate within the co-consumption phase was decreased in the presence of maltose by about 30% (0.50 g/h w/o maltose; 0.35 g/h with maltose) and the maximal L-arabinose consumption rate was decreased by about 20% (0.77 g/h w/o maltose; 0.62 g/h with maltose). As maltose and pentoses do not compete at the transport level and their catabolism converges at the level of phosphofructokinase, these results indicate that the simultaneous catabolism of hexose sugars impedes pentose utilization probably also at the level of glycolytic pathway enzymes.

### The influence of intracellular D-glucose levels and D-glucose phosphorylation on pentose utilization

D-glucose catabolism might impede simultaneous pentose utilization through inhibition by intracellular D-glucose of enzymes of the D-xylose or L-arabinose catabolic pathways. Especially in the case of D-xylose utilization this is not implausible as xylose isomerases also act as *bona fide *glucose isomerases [25]. Moreover, intracellular D-glucose could be transported out of the cells via the hexose transporters [26], and therefore efflux of D-glucose or binding of intracellular D-glucose to the hexose transporters might interfere with pentose uptake.

In order to test possible inhibitory effects, the level of intracellular D-glucose should be raised by eliminating D-glucose phoshorylation. In yeast D-glucose is specifically phosphorylated by hexo- and glucokinases. In contrast, D-xylose must first be converted into D-xylulose which is then phosphorylated by xylulokinase. L-arabinose is first converted into L-ribulose which subsequently is phosphorylated by ribulokinase. Again maltose should be used as the carbon source in order to avoid inhibitory effects of extracellular D-glucose on the uptake of pentoses. Moreover, it has been shown before that hexo-/glucokinase deficient yeast mutants accumulate high intracellular levels of D-glucose when incubated with maltose [27,28].

In *S. cerevisiae *three genes encoding enzymes with hexo-/glucokinase activity are known, *HXK1, HXK2 *and *GLK1 *[29-31]. To block D-glucose phosphorylation all three genes were deleted in the strain BWY1 by using a recyclable *loxP-kanMX-loxP *resistance marker [32], resulting in strain TSY10. The growth properties of TSY10 were characterized on synthetic solid media with various hexoses (D-glucose, D-fructose, D-mannose, D-galactose) as well as maltose or ethanol as carbon sources. TSY10 was also transformed with the plasmids expressing xylose isomerase and xylulokinase, resulting in TSY10-X, and with the plasmids expressing an optimized bacterial L-arabinose catabolic pathway, resulting in TSY10-A, and growth on synthetic solid media with D-xylose and L-arabinose was characterized.

**Figure 2:**
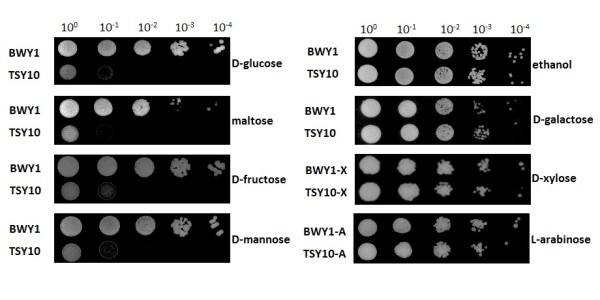
**Growth of *S. cerevisiae hxk/glk*-null strain on different carbon sources**. Cells were spotted in serial dilutions on SC medium agar plates with various carbon sources: 20 g/L D-glucose, 10 g/L maltose, 20 g/L D-fructose, 20 g/L D-mannose, 20 g/L ethanol, 20 g/L D-galactose, 20 g/L D-xylose and 20 g/L L-arabinose. Plates were incubated at 30°C for 2-3 days (D-glucose, maltose, D-fructose, D-mannose, D-galactose), 6 days (ethanol) or 10 days (D-xylose, L-arabinose). BWY1, BWY1-X and BWY1-A served as reference strains.

After incubation for several days at 30°C, no growth could be observed for TSY10 with D-glucose, D-fructose, D-mannose or maltose (Figure 2). As expected, growth on D-galactose or ethanol was identical to the reference strain BWY1 as the hexo-/glucokinases are not involved in the metabolism of these carbon sources [33]. TSY10-X and TSY10-A were still able to grow on D-xylose and L-arabinose, respectively, comparable to BWY1, indicating that pentose consumption is not affected by hexo-/glucokinase deletions (Figure 2).

**Figure 3:**
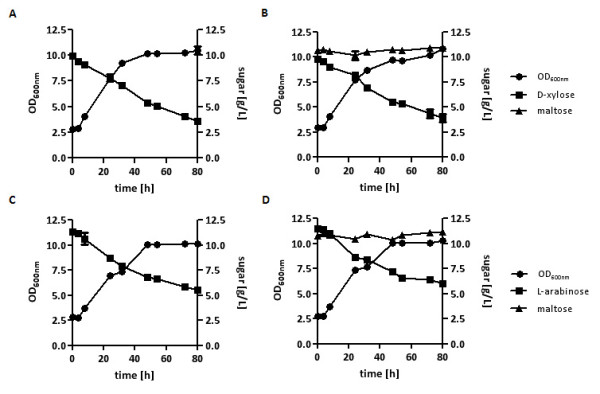
**Analyses of pentose utilization by hexo-/glucokinase deletion strains in the presence of maltose**. The strain TSY10-XT was inoculated with 0.5 g _(dry biomass)_/L in SC medium containing 10 g/L D-xylose (A) or additionally supplemented with 10 g/L maltose (B). The strain TSY10-AT was incubated in SC medium containing 10 g/L L-arabinose (C) or additionally supplemented with 10 g/L maltose (D). The cultivations were performed in shake flasks at 30°C. At different time points, probes were taken, the concentrations of residual sugars analyzed by HPLC, and growth was determined by measuring the OD_600 nm_.

To further increase pentose uptake, TSY10-X was transformed with the plasmid overexpressing *HXT7*, resulting in strain TSY10-XT, and TSY10-A was transformed with the plasmid overexpressing *GAL2*, resulting in strain TSY10-AT. Strains TSY10-XT and TSY10-AT were pregrown in D-xylose- or L-arabinose containing SC media, respectively, and inoculated with about 0.5 g _(dry biomass)_/L in SC media with D-xylose or L-arabinose in the presence or absence of maltose (Figure 3). As expected, due to the deletions of gluco- and hexokinases maltose was not consumed. Growth on pentoses as the sole usable carbon sources and the pentose consumption rates were not affected (less than 5%) by the presence of maltose, indicating that intracellular D-glucose, even at increased levels, has no inhibitory effect on pentose utilization.

### Extracellular D-glucose inhibits pentose consumption

D-xylose is taken up into yeast cells by high- and intermediate-affinity hexose transporters but which have lower affinities for D-xylose than for D-glucose [14,16]. L-arabinose is taken up mainly by the D-galactose transporter Gal2 [21,22], which also transports D-glucose with high affinity [34]. As we already ruled out the possibility that intracellular D-glucose levels impede pentose utilization, we next wanted to test the effect of extracellular D-glucose irrespectively of its further catabolism.

Using the hexo-/glucokinase deletion strains, inhibition of pentose consumption was analyzed by adding increasing amounts of D-glucose to the media. As we observed an incidental occurrence of suppressor mutations enabling cells of the hexo-/glucokinase deletion strain to regain their ability to grow on D-glucose media and to utilize D-glucose as the preferred carbon source low amounts of 2-deoxy-glucose (2-DOG), a phosphorylatable but non-metabolizable analogue of D-glucose [35-37], were added to the growth media. Growth of yeast cells is strongly inhibited by 2-DOG but only after its phosphorylation by hexo-/glucokinases. Addition of 2-DOG should ensure that during long term cultivations cells with suppressor mutations could not further propagate and overgrow the cultures. Indeed, already minor amounts of 2-DOG (0.5 g/L) completely blocked growth of hexo-/glucokinase wild-type strains BWY1-X and BWY1-A on D-xylose and L-arabinose, respectively, whereas they did not affect growth and pentose consumption of the corresponding hexo-/glucokinase deletion strain TSY10-X and TSY10-A. Therefore, in all experiments with D-glucose 0.5 g/L 2-DOG was added.

**Figure 4:**
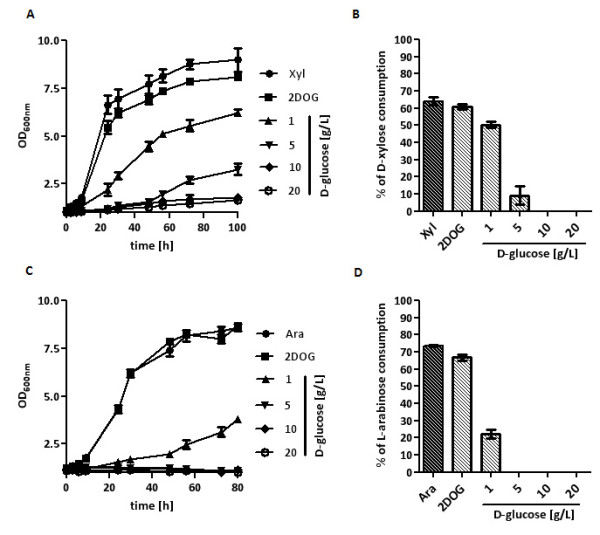
**Pentose utilization by the Δ*hxk/glk*-strain in the presence of different D-glucose concentrations**. The strain TSY10-X was inoculated in shake flasks at 30°C with 0.2 g _(dry biomass)_/L in SC medium with D-xylose (10 g/L) without or with 0,5 g/L 2-DOG (2DOG) and increasing D-glucose concentrations (0 - 20 g/L) in the presence of 2-DOG (0,5 g/L). Growth was determined by measuring the OD_600 nm _(A) and sugar concentrations were analyzed by HPLC. D-xylose consumption at different D-glucose concentrations is displayed as percentage of the total initial D-xylose consumed after 100 h (B). The strain TSY10-A was inoculated in shake flasks at 30°C with 0.2 g _(dry biomass)_/L in SC medium with L-arabinose (10 g/L) without or with 0,5 g/L 2-DOG (2DOG) and increasing D-glucose concentrations (0 - 20 g/L) in the presence of 2-DOG (0.5 g/L). Growth was determined by measuring OD_600 nm _(C) and sugar concentrations were analyzed by HPLC. L-arabinose consumption at different D-glucose concentrations is displayed as percentage of the total initial L-arabinose consumed after 80 h (D).

The strains TSY10-X and TSY10-A were pregrown in pentose media and inoculated (0.2 g _(dry biomass)_/L) in SC media with D-xylose or L-arabinose and increasing amounts of D-glucose. Analysis of growth and sugar consumption showed that with increasing D-glucose concentrations pentose consumption decreased (Figure 4). At low extracellular D-glucose levels (1-5 g/L D-glucose) D-xylose and L-arabinose utilization was inhibited but still possible, while utilization was completely blocked at higher D-glucose concentrations. In all experiments, D-glucose was not consumed at all. The 0.5 g/L 2-DOG did not exert any inhibitory effects.

### Overexpression of hexose transporters improve pentose utilization in the presence of D-glucose

As consumption of pentoses in the presence of low extracellular D-glucose concentrations was possible, we investigated whether overexpression of Hxt7 or Gal2, transporters with the highest capacities for D-xylose and L-arabinose uptake, respectively [12,14,21] might improve pentose utilization in the presence of D-glucose. TSY10-X and TSY10-A were additionally transformed with multicopy vectors expressing *HXT7 *and *GAL2*, respectively, both regulated by strong constitutive promoters.

**Figure 5:**
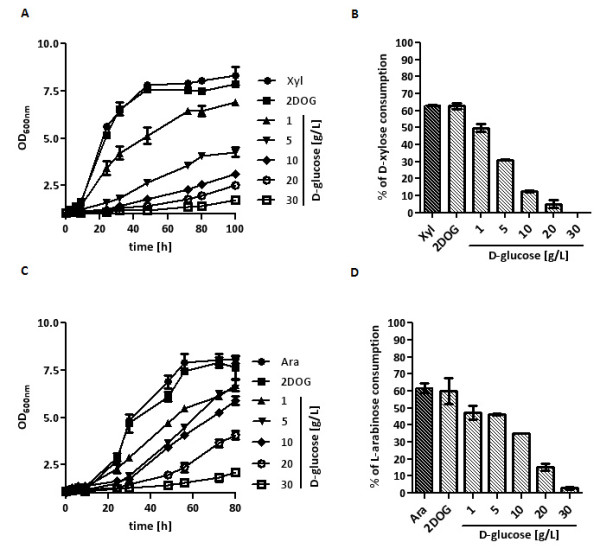
**Pentose utilization by the Δ*hxk/glk*-strain in the presence of different D-glucose concentrations with overexpression of pentose-transporting hexose transporters**. The strain TSY10-XT was inoculated in shake flasks at 30°C with 0.2 g _(dry biomass)_/L in SC medium with D-xylose (10 g/L) without or with 0,5 g/L 2-DOG (2DOG) and increasing D-glucose concentrations (0 - 30 g/L) in the presence of 2-DOG (0.5 g/L). Growth was determined by measuring the OD_600 nm _(A) and sugar concentrations were analyzed by HPLC. D-xylose consumption at different D-glucose concentrations is displayed as percentage of the total initial D-xylose consumed after 100 h (B). The strain TSY10-AT was inoculated in shake flasks at 30°C with 0.2 g _(dry biomass)_/L in SC medium with L-arabinose (10 g/L) without or with 0,5 g/L 2-DOG (2DOG) and increasing D-glucose concentrations (0 - 30 g/L) in the presence of 2-DOG (0.5 g/L). Growth was determined by measuring OD_600 nm _(C) and sugar concentrations were analyzed by HPLC. L-arabinose consumption at different D-glucose concentrations is displayed as percentage of the total initial L-arabinose consumed after 80 h (D).

The resulting TSY10-XT and TSY10-AT strains were pregrown in pentose media and inoculated (0.2 g _(dry biomass)_/L) in SC media containing the corresponding pentoses and increasing D-glucose concentrations. Inhibition of pentose utilization by D-glucose was clearly decreased by overexpression of the transporters (Figure 5) (compare with Figure 4). Residual pentose utilization was still detectable at 20-30 g/L D-glucose, while without overexpression of the transporters pentose consumption was detectable only up to 1-5 g/L D-glucose. Especially, L-arabinose consumption in the presence of higher D-glucose concentrations was improved by overexpression of *GAL2*. D-glucose was not consumed by the strains and supplementation with 0.5 g/L 2-DOG had no influence on pentose utilization.

### Co-consumption of D-xylose and D-glucose

All so far described recombinant pentose utilizing yeast strains consume pentoses much slower than D-glucose which normally is consumed in only a few hours where pentose utilization just begins to become measurable [38-40]. Therefore, in batch cultures it is difficult to analyze whether those strains are able to co-consume the sugars or not. In order to test this in batch cultures, we aimed to decrease D-glucose consumption to levels similar to pentose consumption. For this, we first wanted to stabilize the hexo-/glucokinase deletion strain genetically. In addition to *HXK1, HXK2 *and *GLK1*, one poorly characterized gene, *YLR446W*, exhibiting homologies to hexokinases had been found in the yeast genome sequence [41]. Although our results indicated that this gene is not directly involved in D-glucose phosphorylation we speculated that it might be involved in the occurrence of the incidental suppressor mutations, and therefore we additionally deleted *YLR446W *in strain TSY10. The resulting quadruple deletion strain TSY11 exhibited the same growth and fermentation properties as the triple deletion strain TSY10 (data not shown).

**Figure 6:**
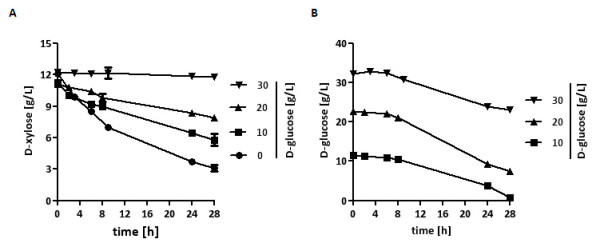
**Xylose-glucose co-consumption**. The strain TSY11-XTH was inoculated in shake flasks at 30°C with 4 g _(dry biomass)_/L in SC medium with D-xylose (10 g/L) and increasing D-glucose concentrations (0-30 g/L). Probes were taken at different time points, and residual D-xylose (A) and D-glucose (B) was analyzed by HPLC.

Then, we re-introduced the *HXK1 *gene on a centromeric plasmid under control of a very weak *UBR2*-promoter [42] into this strain, and additionally overexpressed xylose isomerase, xylulokinase and Hxt7, resulting in strain TSY11-XTH. The strain was pregrown in D-xylose medium and high cell density cultivations (4 g _(dry biomass)_/L) in SC medium containing 10 g/L D-xylose and increasing D-glucose concentrations (0-30 g/L) were performed (Figure 6). About 75% of the initial D-xylose was consumed during 28 hours in the absence of D-glucose, but in the mixed sugar cultivation D-xylose consumption was impaired with increasing amounts of D-glucose. Nevertheless, about 45% of D-xylose was even consumed simultaneously with 10 g/L D-glucose (Figure 6). These results indicate that despite the competition of D-xylose and D-glucose for uptake and further metabolism, D-xylose utilization in the presence of D-glucose and co-consumption of both sugars is possible, at least at the same concentrations of both sugars.

## Discussion

Introduction of pentose catabolic pathways into *S. cerevisiae *enabled this yeast to ferment the lignocellulosic pentoses D-xylose and L-arabinose into ethanol. However, low fermentation rates compared to D-glucose and a lack of pentose utilization in the presence of high D-glucose concentrations are still major obstacles for fermentation of mixed sugar hydrolyzates [38-40]. It is generally assumed that competitive inhibition of pentose uptake by D-glucose is the main problem for simultaneous co-fermentation of D-glucose and pentoses.

To investigate the influence of further D-glucose metabolism on pentose utilization independent of the competition at the uptake level we used the alternative carbon source maltose as a non-competitive transport substrate. Indeed, we found that pentose consumption was impaired by simultaneous maltose utilization (Figure 1). As maltose did not impair pentose utilization in a hexo-/glucokinase mutant strain an inhibitory effect of maltose on pentose uptake can clearly be excluded. Hexose and pentose catabolism converge at the level of phosphofructokinase. Therefore, hexose catabolism might impair pentose utilization on the level of a limiting glycolytic enzyme activity. On the other hand, hexose catabolism could negatively affect regulation of enzymes of the pentose phosphate pathway used for the catabolism of pentoses. Moreover, the results do not exclude the possibility that during the utilization of D-glucose when fluxes are higher than during the utilization of maltose there might be an even stronger impact of D-glucose catabolism on pentose utilization.

Xylose isomerases are well known to also act as *bona fide *glucose isomerases converting D-glucose into D-fructose [25]. Therefore, it might be possible that intracellular D-glucose competes with D-xylose at the level of xylose isomerase. Moreover, it is known that intracellular D-glucose can be transported out of the cells via the hexose transporters [26]. Therefore, increased levels of D-glucose within the cells might negatively influence pentose uptake. In this sense, recently it has been shown that the binding of intracellular D-glucose inhibits the yeast D-glucose sensor Snf3 which has high homologies with the hexose transporters [43]. However, our results with hexo-/glucokinase mutant strains in the presence of maltose could prove that even increased intracellular D-glucose levels did not interfere with pentose isomerization or uptake.

Pentose consumption analyses of the *hxk/glk *null strains confirmed that extracellular D-glucose inhibits pentose consumption in a concentration dependent manner. Increasing concentrations of D-glucose gradually decreased pentose consumption, both in the case of D-xylose as well as L-arabinose (Figure 4). Nevertheless, this experiment and also the co-consumption experiment with the hexokinase-limited mutant strain clearly show that principally co-consumption is possible as long as D-glucose concentrations are low enough.

Accordingly, overexpression of those hexose transporters with a high capacity for D-xylose uptake, Hxt7 [12,14], or L-arabinose uptake, Gal2 [22], partially relieved the inhibitory effects of extracellular D-glucose (Figure 5). Especially in the case of L-arabinose consumption it turned out that constitutive overexpression of *GAL2 *was quite beneficial. This probably is mainly due to the strong repression of the endogenous *GAL2 *gene in the presence of D-glucose [44-46]. Gal2 is the only hexose transporter of *S. cerevisiae *that can effectively transport L-arabinose [21,22]. Therefore, a constitutive overexpession of *GAL2 *is a prerequisite to allow efficient L-arabinose uptake in the presence of D-glucose. In the case of D-xylose, most of the D-xylose-transporting hexose transporters like *HXT7 *are even inducible by low concentrations of D-glucose but are repressed by high concentrations [47,48]. This might explain why D-xylose consumption was less inhibited than L-arabinose consumption by low concentrations of D-glucose as these even induced higher D-xylose transport capacities. Indeed, this would also be in accordance with earlier observations demonstrating that low glucose concentrations (down to 0.1 g/L) increase xylose utilization [49] although we could not observe this with the lowest glucose concentrations used in our study (1 g/L). It was speculated that, beside other effects, increased xylose uptake might be responsible for this.

Our results show that co-fermentation of D-glucose and pentoses can be improved either by keeping D-glucose concentrations on a low level or by the expression of specific heterologous pentose transporters that are not inhibited by D-glucose. Indeed, using prefermentation and fed-batch systems to minimize initial D-glucose concentrations D-xylose utilization could recently be increased in a simultaneous saccharification and co-fermentation (SSCF) process [50,51]. Moreover, in *E. coli *co-consumption of D-glucose and D-xylose could be demonstrated just by eliminating catabolite repression by D-glucose of D-xylose specific transporters xylE or xylFGH [52,53]. On the other hand, Ha et al. [54] recently engineered a xylose fermenting *S. cerevisiae *strain for simultaneous fermentation of xylose and cellobiose by expressing a specific cellobiose transporter together with an intracellular β-glucosidase.

For future perspective, a pentose-fermenting hexo-/glucokinase deletion strain might be an interesting screening system to select for mutants able to ferment pentoses in the presence of increasing concentrations of D-glucose, e.g. via evolutionary engineering [55]. Reintroduction of hexokinase activity should then result in strains able to consume pentoses even in the presence of higher concentrations of D-glucose.

## Conclusions

Our results suggest that co-fermentation of pentoses in the presence of D-glucose can significantly be improved by the overexpression of pentose transporters, especially if they are specific for the pentoses and not inhibited by D-glucose. On the other hand, this could lead to other limitations further downstream in the co-metabolism of both kind of sugars.

## Methods

### Strains and media

Yeast strains and plasmids used in this work are listed in Table 1.

**Table 1:** *S.cerevisiae *strains and plasmids used in this study

*S. cerevisiae *strain or plasmid	Relevant genotype or phenotype	Source or reference
**Strains**		
CEN.PK2-1 C	*MAT*a *leu2-3*,*112 ura3-52 trp1-289 his3-*_*1 MAL2-8*c *SUC2*	EUROSCARF, Frankfurt
BWY1	*MAT*a *leu2-3*,*112 ura3-52 trp1-289 his3-*_*1 MAL2-8*c *SUC2*; unknown beneficial mutations for L-arabinose utilization	[11]
BWY1-X	BWY1 with plasmids YEplac181_opt.Xi, p423pPGK1-XKS Tr	This work
BWY1-A	BWY1 with plasmids p423H7-synthIso, p424H7-synthKin, p425H7-synthEpi	This work
BWY1-XT	BWY1 with plasmids YEplac181_opt.Xi, p423pPGK1-XKS Tr, p426_*HXT7*	This work
BWY1-AT	BWY1 with plasmids p423H7-synthIso, p424H7-synthKin, p425H7-synthEpi and pHL125^re^	This work
TSY10	*MAT*a *leu2-3*,*112 ura3-52 trp1-289 his3-*_*1 MAL2-8*c *SUC2 *Δ*hxk1::loxP* Δ*glk1::loxP *Δ*hxk2::loxP*; unknown beneficial mutations for L-arabinose utilization	This work
TSY10-X	TSY10 with plasmids YEp181_pHXT7-optXI_Clos and p423pPGK1-XKS Tr	This work
TSY10-XT	TSY10 with plasmids YEp181_pHXT7-optXI_Clos, p423pPGK1-XKS Tr and p426_*HXT7*	This work
TSY10-A	TSY10 with plasmids p423H7-synthIso, p424H7-synthKin and p425H7-synthEpi	This work
TSY10-AT	TSY10 with plasmids p423H7-synthIso, p424H7-synthKin, p425H7-synthEpi and pHL125^re^	This work
TSY11	*MAT*a *leu2-3*,*112 ura3-52 trp1-289 his3-*_*1 MAL2-8*c *SUC2 *Δ*hxk1::loxP* Δ*glk1::loxP *Δ*hxk2::loxP *Δ*ylr446w::loxp-kanMX-loxp*; unknown beneficial mutations for L-arabinose utilization	This work
TSY11-XTH	TSY11 with plasmids YEp181_pHXT7-optXI_Clos, p423pPGK1-XKS Tr, p426_*HXT7 *and pRS314p*UBR2-HXK1*	This work
**Plasmids**		
p426H7	2 μ-plasmid; *URA3 *marker gene	[10]
YEpk*HXT7*	DNA-template for amplification of *HXT7*	[61]
p426_*HXT7*	2 μ-plasmid*; HXT7 *under control of shortened *HXT7*-promoter and *CYC1*-terminator*; URA3 *marker gene	This work
pHL125^re^	2 μ-plasmid; *GAL2 *under control of *ADH1-*pomoter, *URA3 *marker gene	[62]
p423H7-synthIso	2 μ-plasmid; codon-optimized *araA *of *B. licheniformis *under control of shortened *HXT7*-promoter and *CYC1*-terminator*; HIS3 *marker gene	[11]
p424H7-synthKin	2 μ-plasmid; codon-optimized *araB *of *E. coli *under control of shortened *HXT7*-promoter and *CYC1*-terminator*; LEU2 *marker gene	[11]
p425H7-synthEpi	2 μ-plasmid; codon-optimized *araD *of *E. coli *under control of shortened *HXT7*-promoter and *CYC1*-terminator*; TRP1 *marker gene	[11]
pUG6	DNA-template for amplification of loxP-*kanMX-loxP *gene resistance marker	[32]
pSH47	Cre-recombinase under control of *GAL1 *promoter*; URA3 *marker gene	[32]
YEp181_pHXT7-optXI_Clos	2 μ-plasmid; codon-optimized xylose isomerase gene of *C. phytofermentans *under control of shortened *HXT7*-promoter and *CYC1*-terminator; *LEU2 *marker gene	Boles, lab stock
p423pPGK1-XKS Tr	2 μ-plasmid; xylulokinase gene of *H. jecorina *under control of *PGK1*-promoter and *CYC1*-terminator*; HIS3 *marker gene	Boles, lab stock
pRS314	Yeast centromere vector; *TRP1 *marker gene	P. Kötter, Frankfurt, Germany
pRS314p*UBR2-HXK1*	Yeast centromere vector; *HXK1 *of *S. cerevisiae *behind *UBR2*-promoter and *HXK1*-terminator; *TRP1 *maker gene	This work

*S. cerevisiae *was grown in synthetic complete (SC) medium (1.7 g/L Difco yeast nitrogen base without amino acids and 5 g/L ammoniumsulfate), supplemented with amino acids but omitting the selective plasmid markers nutrients as described previously [56], containing various carbon sources.

For serial dilution growth assays, cells growing in exponential phase were collected and resuspended in sterile water to an OD_600 nm _of 1. Cells were serially diluted in 10-fold steps, and 5 μl of each dilution was spotted on agar plates. In aerobic batch cultivations, *S. cerevisiae *was grown in SC medium supplemented with maltose, D-glucose, D-xylose or L-arabinose as carbon sources and buffered at pH 6.3 with 20 mM KH_2_PO_4_. Plasmids were amplified in *Escherichia coli *strain DH5α (Gibco BRL, Gaithersburg, MD) or strain SURE (Stratagene, La Jolla, CA). *E. coli *transformations were performed via electroporation according to the methods of Dower et al., 1988 [57]. *E. coli *was grown on LB (Luria-Bertani) medium with 40 μg/ml ampicillin for plasmid selection.

### Construction of *hxk*-null strains

Strains lacking hexo-/glucokinase genes were constructed employing the *loxP:: kanMX:: loxP*/Cre recombinase system and the 'short flanking homology PCR' technology [32]. The primers used for the construction of the replacement PCR constructs (obtained from biomers.net) are listed in Table 2.

**Table 2:** Primers used for gene deletions

***Δhxk1for***	5'-**ATGGTTCATTTAGGTCCAAAGAAACCACAGGCTAGAAAGGGTTCCATGGC***TTCGTACGCTGCAGGTCGAC*-3'
***Δhxk1rev***	5'-**TTAAGCGCCAATGATACCAAGAGACTTACCTTCGGCAATTCTTTTTTCGG***GCATAGGCCACTAGTGGATCTG*-3'
***Δglk1for***	5'-**ATGTCATTCGACGACTTACACAAAGCCACTGAGAGAGCGGTCATCCAGGC***TTCGTACGCTGCAGGTCGAC*-3'
***Δglk1rev***	5'-**TCATGCTACAAGCGCACACAAGGCGGCACCCACTCCGGAACCATCCTTGG***GCATAGGCCACTAGTGGATCTG*-3'
***Δhxk2for***	5'-**ATGGTTCATTTAGGTCCAAAAAAACCACAAGCCAGAAAGGGTTCCATGGC***TTCGTACGCTGCAGGTCGAC*-3'
***Δhxk2rev***	5'-**TTAAGCACCGATGATACCAACGGACTTACCTTCAGCAATTCTTTTTTGGG***GCATAGGCCACTAGTGGATCTG*-3'
***Δylr446wfor***	5'-**ATGACAATTGAAAGCACTCTAGCTCGGGAATTAGAAAGCTTGATTTTACC***TTCGTACGCTGCAGGTCGAC*-3'
***Δylr446wrev***	5'-**TTATTGAACTTGGTTGTCTGATTTGTTCAAGTAGGTGGCTATTGCAGCGCC***GCATAGGCCACTAGTGGATCTG*-3'

Homology to the hexo-/glucokinase genes, bold; homology to *kanMX *cassette, italic.

Yeast transformations were carried out as described previously [58,59]. As induction of the D-galactose-inducible, D-glucose-repressible Cre recombinase on plasmid pSH47 by D-galactose appeared to have deleterious effects on cells containing several *loxP *sites, we routinely used maltose (which has a weaker repressive effect than D-glucose) to induce/derepress *loxP*-Cre recombination. The hexokinase genes were deleted successively in the following order: *HXK1, GLK1, HXK2 *and *YLR446W *selecting for G418 resistance on yeast extract-peptone medium with 10 g/L maltose or 20 g/L ethanol (after deletion of *GLK1*).

### Construction of plasmids

The coding region of *HXT7 *from *S. cerevisiae *was amplified by PCR from YEpk*HXT7 *and cloned into the linearized vector p426H7 (*URA3*) by recombination cloning, employing the procedure already used in Wieczorke et al. (29) and omitting the six histidine codons. The coding region of *HXK1 *with 300 bp downstream of the open reading frame was amplified with primers (HXK1_for_UBR2: 5'-TAGCTACTTAACAAGCACGCATGGTTCATTTAGGTCCAAAGAAACCACAGGC-3'; HXK1_Term_rev: 5'-GAAAAACCGTCTATCAGGGCGATGGCCCACTACGTGAACCATCACCAAAGCAATGGATTATGCCATAAG-3') from genomic DNA of CEN.PK2-1 C with homologous regions to the pRS314 vector at its 3'-end and to the *UBR2*-promoter at its 5'-end. For the *UBR2*-promoter a fragment of 500 bp upstream of the *UBR2*-open reading frame was amplified with primers (UBR2_Prom_for**: **5'- GTGGAATTGTGAGCGGATAACAATTTCACACAGGAAACAGCTATCCCCCGTTTAGAGGAAGG -3'; UBR2_Prom_rev: 5'-GCCTGTGGTTTCTTTGGACCTAAATGAACCATGCGTGCTTGTTAAGTAGCTA -3') from genomic DNA of CEN.PK2-1 C with homologous regions to the open reading frame of *HXK1 *and to the pRS314 vector. Both fragments were fused by cloning them into the linearized (*Bam*HI, *Xho*I) pRS314 vector by recombination cloning. Yeast transformations of plasmid DNA from yeast cells were carried out as described above [58,59]. Molecular techniques were performed according to published procedures [60].

### Growth assays

Cultures (50 ml) were grown in 300-ml flasks at 30°C with constant shaking at 180 rpm. Precultures were grown in SC medium containing 20 g/L L-arabinose or 20 g/L D-xylose. Cells were washed with sterile water and inoculated in SC medium containing various combinations of carbon sources (L-arabinose, D-xylose, D-glucose, D-maltose, 2-deoxy-glucose). Dry biomass was determined by filtering 10 ml of the culture through a pre-weighted nitrocellulose filter (0.45 μm pore size; Roth, Germany). The filters were washed with demineralized water, dried in a microwave oven for 20 minutes at 140 W, and weighted again. All growth assays and sugar consumption analyses were carried out at least in duplicate.

### Sugar analyses

The concentrations of sugars were determined by high-performance liquid chromatography (Dionex BioLC) using a Nugleogel Sugar 810 H exchange column (Macherey-Nagel GmbH & Co, Germany). The column was eluted at the temperature of 65°C with 5 mM H_2_SO_4 _as a mobile phase with a flow rate of 0.6 ml/min. Detection was done by means of a Shodex RI-101 refractive-index detector. Chromeleon software (version 6.50) was used for data evaluation.

## Competing interests

The authors declare competing financial interests. EB is co-founder of the Swiss biotech company Butalco GmbH.

## Authors' contributions

TS designed and performed the experiments and wrote the first draft of the manuscript. EB initiated this work, contributed to experimental design and edited the final manuscript. All authors read and approved the final manuscript.
